# Evaluation of tribo-mechanical measurements and thermal expansion of Cu-based nanocomposites reinforced by high strength hybrid ceramics

**DOI:** 10.1038/s41598-024-67173-9

**Published:** 2024-07-30

**Authors:** Mai Z. Zaki, M. M. El-Zaidia, H. M. Abomostafa, Mohammed A. Taha

**Affiliations:** 1 Department of Basic Science, Higher Institute of Engineering and Technology, Menoufia, Egypt; 2 Faculty of Science, Physics Department, Menoufa University, Shebin Elkom, Egypt; 3 Solid State Physics Department, National Research Centre, Dokki, 12622 Cairo Egypt; 4 Pharos University in Alexandria, Canal Mahmoudiah Street, Smouha, Alexandria Egypt

**Keywords:** Cu matrix, Hybrid nanocomposites, Graphene, Wear resistance, Powder metallurgy, Mechanical properties, Materials science, Nanoscale materials

## Abstract

It is known that Copper’s (Cu) electrical conductivity makes it a desirable material for use in industry. Due to poor properties such as hardness, thermal expansion, and corrosion resistance, its applications are limited. This manuscript solves these problems while maintaining no breakdown in electrical conductivity. In this study, high-strength ceramics (SiC nanoparticles and graphene nanosheets) were used as reinforcements in the manufacture of Cu-based hybrid nanocomposites using powder metallurgy technique. X-ray diffraction analysis (XRD) was used to investigate phase composition and crystal size of the milled powders. Transmission electron microscopy (TEM) and field emission scanning electron microscopy (FESEM), respectively examined the microstructure of the prepared powder powders and sintered nanocomposites. Then, various properties of the sintered samples are measured, including physical, electrical and thermal properties and wear resistance. The obtained XRD technique and TEM images showed decreases in the crystal and particle size of milled samples reaching up to 14.08 and 28.30 nm, respectively for the sample contained 8 vol. % SiC + 0.8 vol. % graphene (SG8). A surprising improvement in the mechanical properties of up to 809.15, 341.84 MPa and 336.56 GPa for microhardness, strength and longitudinal modulus for the sample containing the highest reinforcements, achieving an improvement of up to 122, 61.37 and 41 percent compared to the Cu matrix. Moreover, there was a noticeable improvement in the coefficient of thermal expansion (CTE) and wear rate values of the samples by increasing the percentages of hybrid reinforcements in the examined sintered nanocomposite samples. The Sample SG8 recorded the lowest value, decreasing by about 50.2 and 76.5% compared to the SG1 sample. Finally, adding reinforcements to the Cu matrix had a negative effect on the relative density and electrical conductivity, and the lowest values was 92.94% and8.59 × 10^6^ S/m, respectively for the SG sample.

## Introduction

There is an urgent need to produce high-efficiency metals due to the weakness of some of the properties of the metals currently used, which limits their widespread use in industrial applications. Cu has been widely used in high-end application fields, particularly in power generation, electronics industries, rail transit, and others, because of its unique properties, which include good thermal and electrical conductivities. However, these uses are limited by its low strength, low hardness, CTE, and wear resistance^1–3^. Accordingly, it is possible to improve the aforementioned properties by adding mono ceramic particles and producing composite materials with a Cu matri such as TiO_2_^4^, Al_2_O_3_^5^, B_4_C^6^, graphene^7^, Si_3_N_4_^8^. SiC^9^ nanoparticles are a type of reinforcement that is added to the Cu matrix because of their low cost, high hardness, high thermal conductivity, good wear, and low CTE. These properties allow the SiC particles to significantly increase the strength and wear properties without severely compromising the electrical and thermal properties of the Cu^10,11^. Graphene nanosheets is regarded as the best option for enhancing the characteristics of Cu because of its exceptional mechanical and electrical qualities as well as its light weight. Consequently, graphene presents a suitable reinforcement for the creation and investigation of Cu matrix composites^12,13^. It is important to note, that the mechanical properties, CTE and wear resistance of the nanocomposites produced are further improved by the small grain size of the matrix and the uniform distribution of nano-reinforcements. Moreover, the amount of added reinforcement and its smaller size have a positive effect on improving the aforementioned properties. Hybrid Cu-based composites are manufactured composite materials with more than one type of reinforcement that can produce a satisfactory result. As a result, there are always many attempts by researchers to produce Cu base nanocomposites that have distinctive properties that can be widely used in previous applications. For example, Cu–ZrB_2_ composites were made by Fan et al.^14^ using the melting-casting process. They demonstrated that the hardness and wear resistance of the composites enhanced with an increase in ZrB_2_ content. The composites’ electrical conductivity dropped at the same period. Guo et al.^15^ extensively studied the effect of 12 volume percent graphene on mechanical properties of Cu matrix. Their results showed an improvement in strength of about 40% compared to Cu. Singh et al.^16^ used a stir casting manufacturing technique to create copper metal matrix composites reinforced with B_4_C, TiC and BN. Composites’ densities, tensile and compressive strengths, hardness, and electrical conductivity were all examined. It is discovered that reinforced composites outperform pure Cu in terms of mechanical properties. As the amount of reinforcement in composite increases, its density and electrical conductivity drops.

Samani et al.^10^ prepared Cu matrix composites reinforced with various content of SiC. They found an increase in microhardness and ultimate strength of about 27 and 17%, respectively, compared to that of pure Cu. It is worth noting that nanocomposites improved with a single ceramic reinforcement focused only on improving one or two properties at most, and at the same time, it was possible to improve the required properties to an insufficient degree. In many cases, it is not desirable to add a large amount of ceramic to the copper matrix because it has many disadvantages, such as the negative effect on the rest of the desired properties and the difficulty of preparation, because the greater the amount of ceramic, the more difficult it is to distribute it homogeneously^12^. New materials were produced by adding hybrid particles in various proportions to the metal matrix. Undoubtedly, using hybrid reinforcements in a metal matrix leads to more improvement in microhardness, Young’s modulus, strength, wear resistance, and CTE value compared to using single reinforcement^17^.

In general, there are different techniques commonly used to produce metal-based nanocomposites, including stir casting^18,19^, fracture stirring^2,20^ and powder metallurgy (PM)^12,21^. PM is a cost-effective method for the mass production of metal matrix nanocomposites. PM provides a number of important benefits, such as improved control over reinforcement distribution and creation of a more uniform matrix microstructure, which decreases segregations. Through a series of cold welding, fracturing, and re-welding steps in a high-energy ball mill, powder particles may be dispersed throughout the metal matrix in this powder processing procedure. Because of the mechanical forces at play and the particles’ heightened stress from repeated work, the powder will be cold welded together^22–24^.

There is research that uses SiC nanoparticles or graphene nanosheets to improve the mechanical properties and wear resistance of copper, but with high reinforcement ratios, sometimes reaching 8 vol. % Gr and 30 vol. % SiC to achieve the desired improvement, whether in mechanical properties or wear resistance, but increasing the amount of reinforcement has a negative effect on other properties, especially physical and electrical properties. The novelty of this work is the use of a hybrid of SiC nanoparticles and graphene nanosheets as reinforcements in appropriate proportions of up to 8 and 0.8 vol. %, respectively, to improve the aforementioned properties of copper using PM technique to obtain nanocomposites that have mechanical properties, wear resistance, and a and a low CTE value. In addition, it maintains no significant decrease in electrical conductivity, so it can be widely used in the various industrial applications previously mentioned. The novelty of this work is the use of powder metallurgy technology to obtain hybrid nanocomposites based on Cu and enhanced with different proportions of SiC up to 8 vol. % and graphene with proportions up to 0.8 vol. % then pressing and sintered for 1 h at temperatures of 700, 800, and 850 °C in an argon atmosphere. Moreover, the effect of adding hybrid reinforcement on microstructure and various properties, including density, mechanical properties, CTE value, wear resistance, and electrical conductivity of prepared nanocomposites, was investigated.

## Experimental procedure

### Preparation of nanocomposites powders

In this work, Cu (≈ 100 μm) was chosen as the matrix, while the as-prepared SiC nanoparticle and grapheme nanosheet were used as reinforcements. The chemical compositions of the batch compositions designed for nanocomposites with Cu matrix, along with their abbreviations, are tabulated in Table 1. Each sample was milled for 20 h at 440 rpm, BPR was 15:1, and 0.1% stearic acid was added. It is important to note that the milling was completed in five hours with a two-hour break.

**Table 1:** Scheme of the prepared composites referring to the sample name and its composition (vol. %).

Sample name	Composition (vol. %)		
	Cu	SiC	Graphene
SG0	100	–	–
SG1	98.9	1	0.1
SG2	97.8	2	0.2
SG4	95.6	4	0.4
SG8	91.2	8	0.8

### Characterization of the prepared powders

X-ray diffraction (XRD; Philips PW) technique was used to detect the phases of the milled powders. X-ray line broadening for the principle (hkl) planes (1 1 1, 2 0 0 and 2 2 0) was used to calculate the crystalline size (D), lattice strain (ε), and dislocation density ($\updelta$) using the formulae described in Ref^25^:

1 $${\text{D}} = \frac{0.9\lambda }{{{\text{B}}\cos \theta }}$$

2 $$\varepsilon = \frac{{\text{B}}}{4\tan \theta }$$

3 $$\delta = \frac{1}{{D^{2} }}$$

Transmission electron microscopy (TEM, model JEOL JEM-1230) was used to analyze the morphology and particle sizes of the mechanically milled powders.

### Characterization of the sintered nanocomposites

The milled powders were pressed by a hydraulic press using a load of 30 MPa, After that, the sintering process was carried out at a temperature of 700, 800 and 850 °C in argon gas for 1 h and a heating rate of 5 °C/min.

#### Physical properties

The archimedes method (ASTM: B962-13) was used to study the relative density and apparent porosity of nanocomposites at different sintering temperature, as detailed in our recent work^26,27^.

#### FHSEM investigation

FHSEM coupled with energy dispersive X-ray analysis **(**EDX) (type Quanta FEG250 with an accelerating voltage of 30 kV and a magnification of 10 × up to × 300,000) was used to examine their microstructure.

#### Thermal properties

Thermal expansion of nanocomposites samples was measured from 30 up to 500 °C using an automatic Netzsch DIL402 PC (Germany) with a heating rate of 5 °C min^−1^using rectangular bars.

#### Mechanical properties

As reported in our recent work, the Vickers tester was used to determine the microhardness (Hv) of the sintered samples in accordance with ASTM: B933–09, with an applied stress of 1.9 N for 10 s^28,29^.

4 $${\text{Hv}} = 1.854{ } \times { }\frac{{\text{P}}}{{{\text{d}}^{2} }}$$

where P is applied load (1.9 N) and d is the diagonal of indentation.

A hydraulic machine examined the ultimate strength according to ASTM E9.

Ultrasonic longitudinal (V_L_) and shear wave velocities (V_S_) were measured in the nanocomposites, using the pulse-echo technique system. The values of Lame’s constants are obtained from V _L_ and V_S_ as follows^30,31^:

5 $$\lambda = \rho \left( {{\text{V}}_{L}^{2} - 2{\text{V}}_{S}^{2} } \right)$$

6 $$\mu = \rho V_{S}^{2}$$

The longitudinal modulus (L), Young’s modulus (Y), shear modulus (G), bulk modulus (B) and Poisson’s ratio (ν) of the nanocomposite were calculated according to the formula^32,33^:

7 $$L = \lambda + 2\mu$$

8 $$G = \mu$$

9 $$E = \mu \frac{3\lambda + 2\mu }{{\lambda + \mu }}$$

10 $$B = \lambda + \frac{2}{3}\mu$$

11 $$\upsilon = \frac{\lambda }{{2\left( {\lambda + \mu } \right)}}$$

#### Wear test

The wear rate (W) measure was calculated by the following Eqs. (12) and (13) using a pin-on-disc wear-testing apparatus^34^. The wear test process conditions included a speed of 0.8 m/s, a time 10 min, and applied loads of 10, 20, and 40 N.

12 $${\text{Net weight}} = {\text{weight before wear}} - {\text{weight after wear}}$$

13 $${\text{wear rate}} = \frac{{\text{Net weight}}}{{\text{Sliding time}}}$$

#### Electrical conductivity

The electrical conductivity (σ) of the nanocomposites was measured e using Keithley device according to the formula^35^:

14 $$\sigma = \frac{{\text{h}}}{{{\text{RA}}}}$$

where R, h and A are the electrical resistance, the sample diameter and the sample surface area.

## Results and discussion

### X-ray analysis

**Figure 1:**
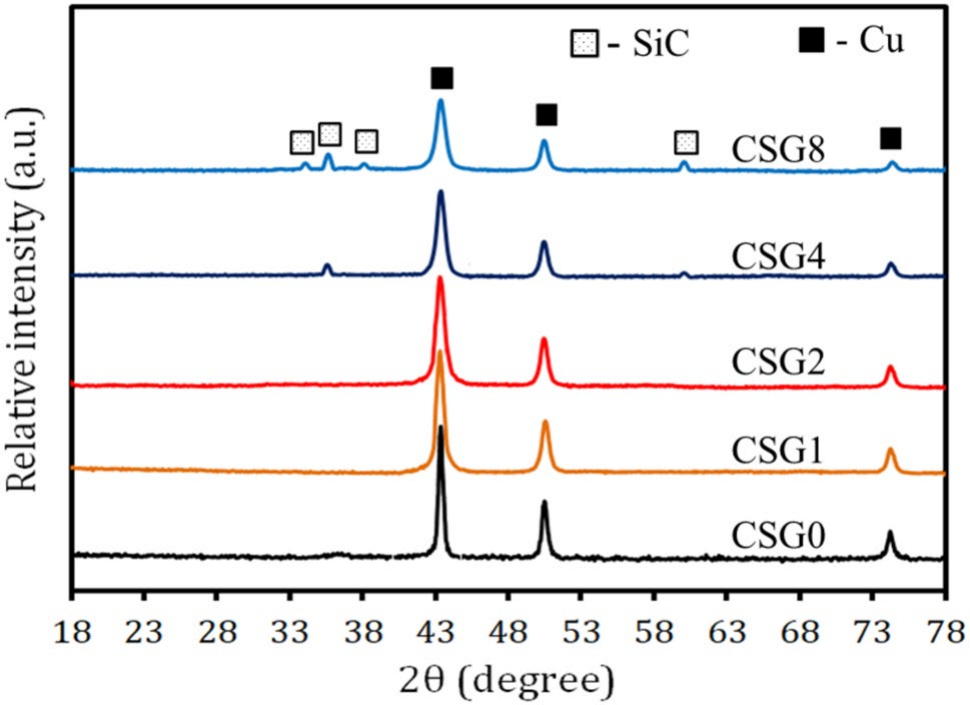
The XRD patterns of the SG0, SG1, SG2, SG4, and SG8 milled powders.

**Figure 2:**
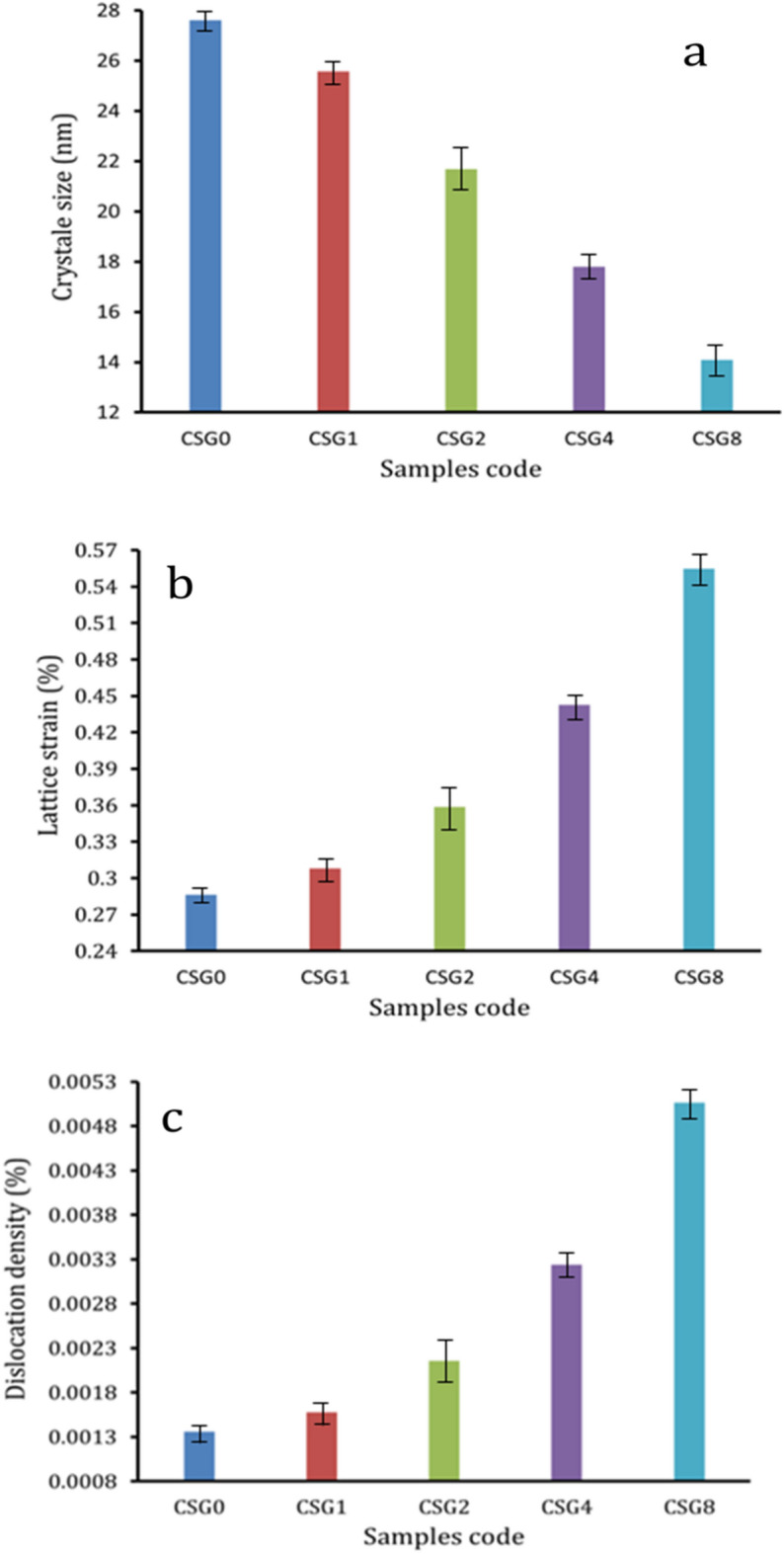
Effect of adding hybrid reinforcements on the, (**a**) crystal size, (**b**) lattice strain, and (**c**) dislocation density of milled powders.

The XRD patterns of powdered of milled Cu hybrid nanocomposites contain SiC nanoparticles and graphene nanosheets concentrations are shown in Fig. 1. According to the representation, only two phases (i.e. Cu and SiC) appear to have developed. These phases correspond to JCPDS-ICDD card numbers 85-1326 and 89-2225, respectively. Because the volume percentages of grapheme are small (≥ 0.8 vol. %) and also SiC in the SG1 and SG2 samples (1 and 2 vol. %, respectively) are below the XRD detection limit, there are no peaks representing the reinforcement^36^. Figure 2 shows how D, Ɛ, and δ change depending on the makeup of the milled powders. It is shown that the inclusion of both hard ceramic reinforcements, which function as milling balls and transmit more energy to the Cu matrix, causes the crystallite size to drop as SiC and graphene contents rise while lattice strain and dislocation density increase. Moreover, it has been demonstrated that because of extreme plastic deformation and grain size refinement during the milling process, the intensity of the peaks decreases and broadens as the volume percentage of ceramics reinforcements increases^37^.

### TEM observations

**Figure 3:**
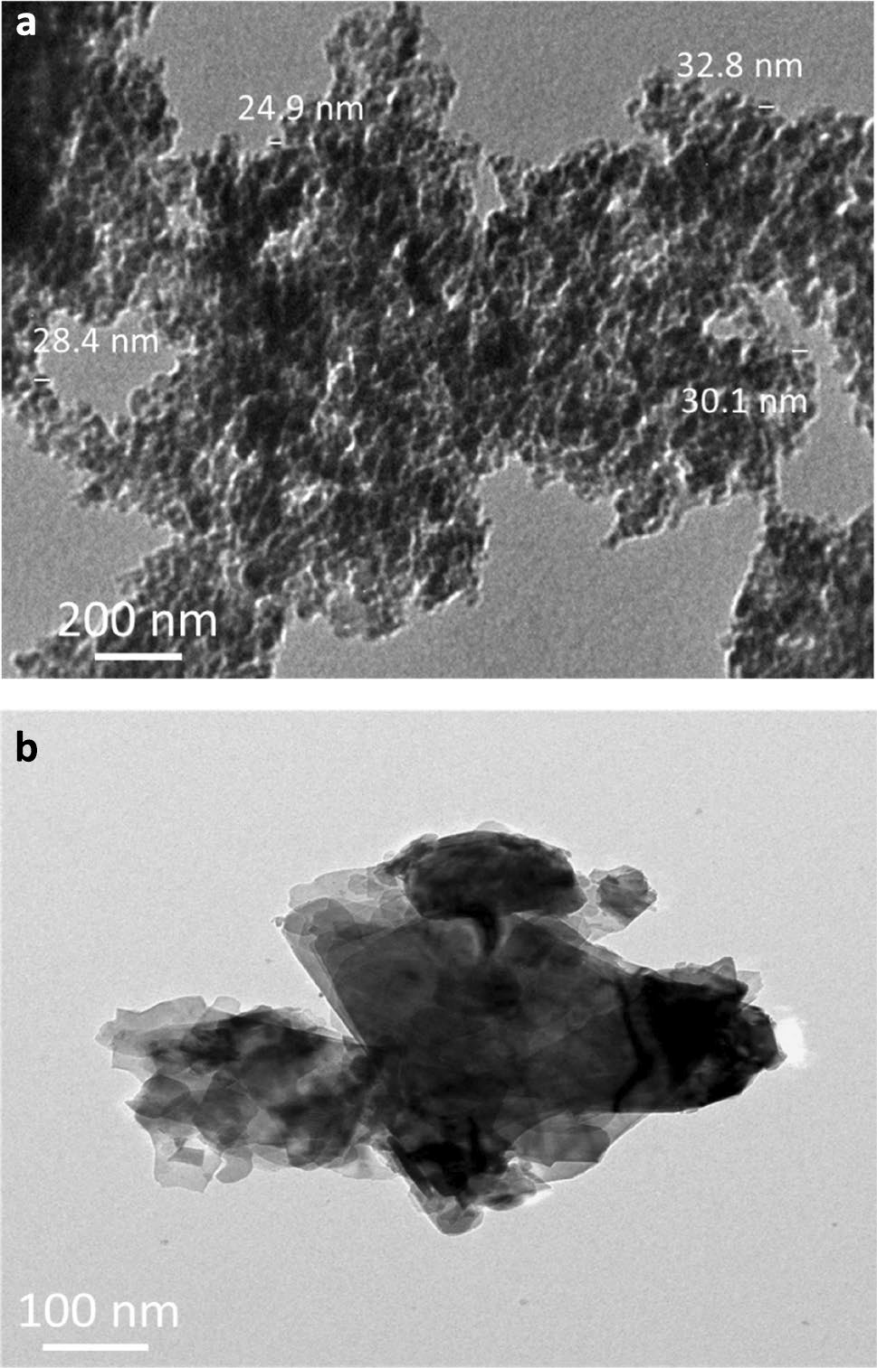
TEM images of (**a**) SiC nanoparticles and (**b**) graphene nanosheets.

**Figure 4:**
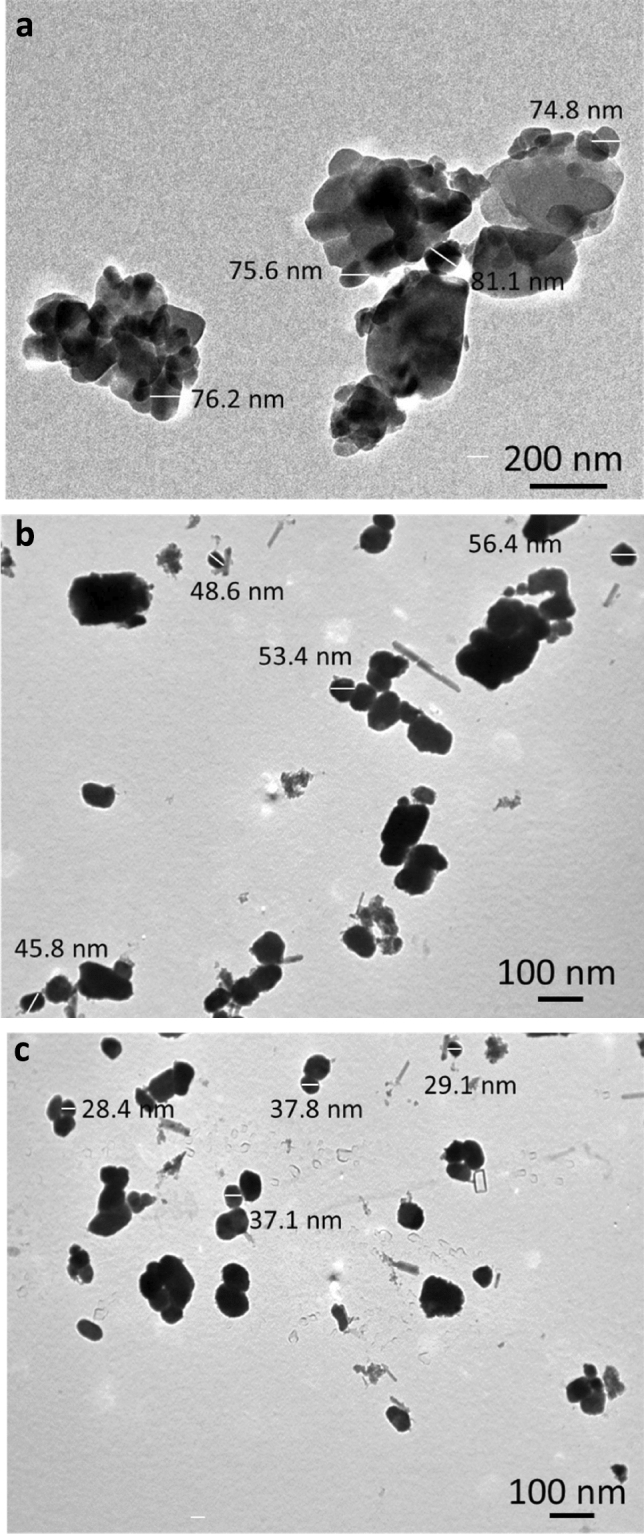
TEM images of SG0, SG4 and SG8 samples samples.

**Figure 5:**
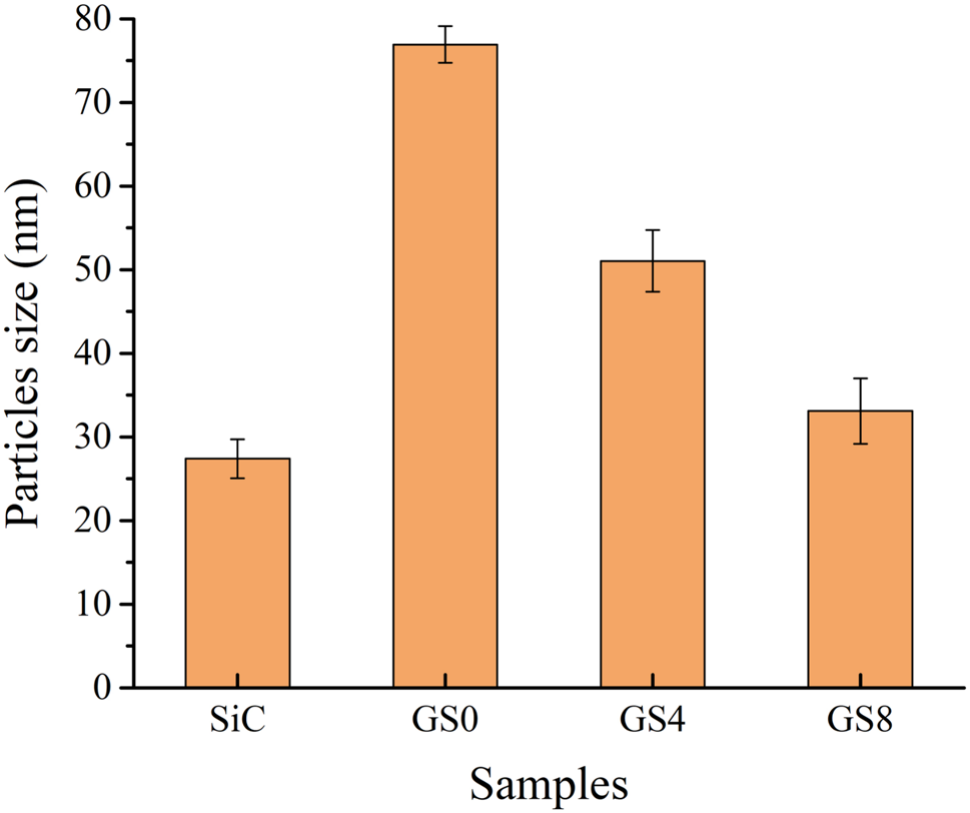
Average particle size of SiC, GS0, GS4, and GS8 samples.

Figure 3a,b shows TEM photos of SiC nanoparticles and graphene nanosheets reinforcements. The graphene powder appears in the form of sheets, while the SiC powder appears in the form of particles with a size of 27.4 nm. TEM images of powdered milled copper and its hybrid nanocomposites are shown in (Fig. 4a–c). The SG0 sample, as shown in (Fig. 4a), clusters together due to its flexibility and its 76.9 nm particle size. In contrast, the agglomeration has somewhat diminished, and the particle sizes of the SG4 and SG8 samples are 51.03 and 33.1 nm, respectively, in (Fig. 4b,c). Figure 5 shows the effect of added hybrid ceramic particles on the particle size of the Cu matrix. Generally, the ductile particles (Cu) suffer from deformation, and the brittle ones (SiC and graphene) suffer from fragmentation. Therefore, at the beginning of the milling process, the Cu particles start to weld, while the SiC and graphene ones come between two or more Cu particles. This means that the fragmented ceramic particles stay at the interfacial boundaries of the welded Cu particles, and consequently, the nanocomposite powders are actually formed with reduced particle size.

### Relative density and apparent porosity

**Figure 6:**
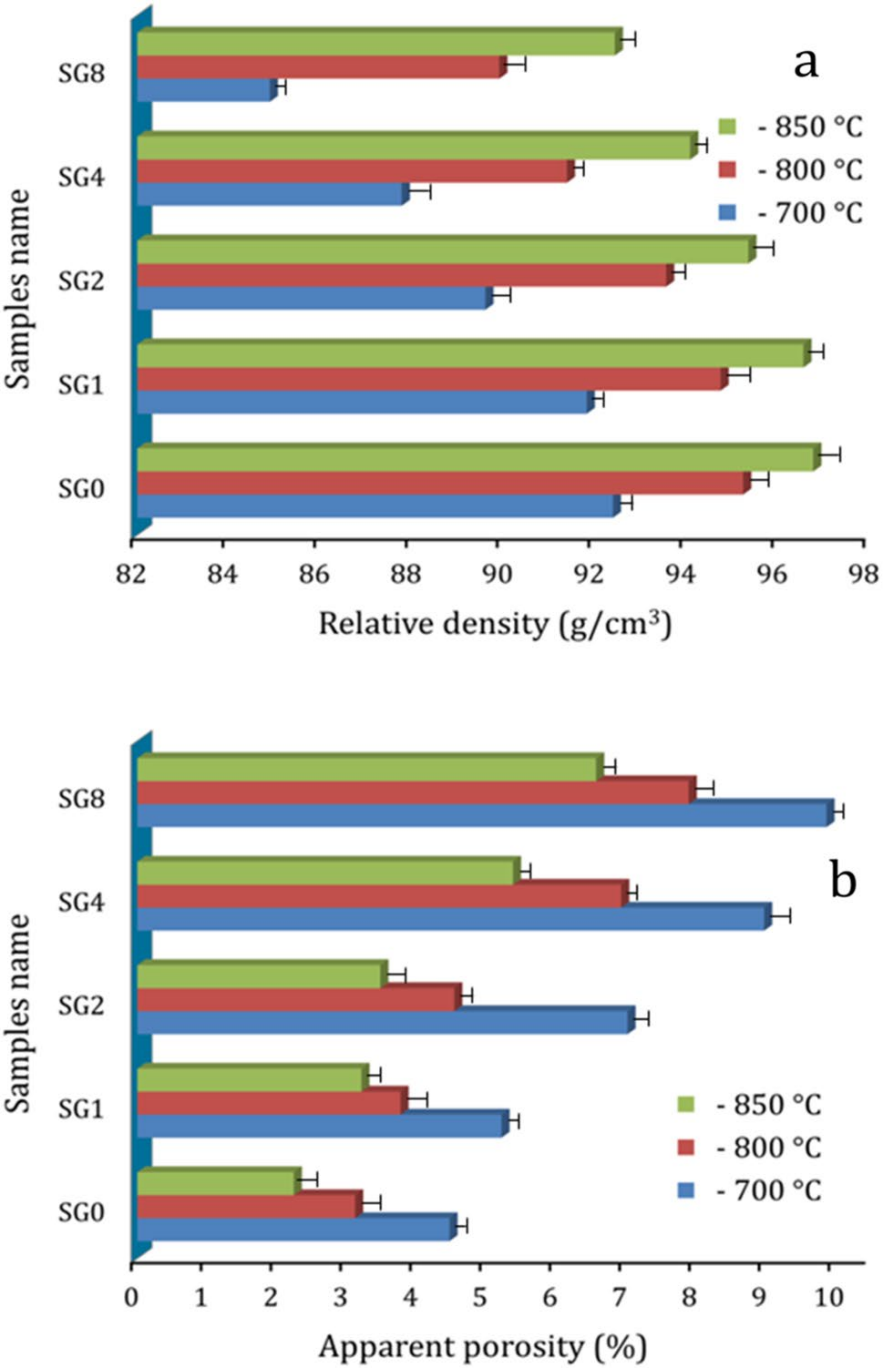
(**a**) Relative density, and (**b**) apparent porosity of samples at different sintering temperatures.

The effect of sintering temperature and adding different volume contents of hybrid ceramics on the relative density and apparent porosity of the samples was measured and shown in Fig. 6 (a,b). It is evident that a rise in the volume percentage of ceramics in the nanocomposites causes the specimens' apparent porosity to increase and their densities to decrease. This outcome might be the result of the greater hardness of the reinforcement particles in the Cu matrix decreasing the pressing capacity of the sintered samples as the reinforcement contents increase. This result can be explained by knowing that the density of SiC and graphene (3.21 and 2.27 g/cm^3^, respectively) is more than half that of Cu matrix (8.96 g/cm^3^). Therefore, replacing a heavier element with a lighter heavier one leads to decreased densities and an increase the apparent porosity of sintered samples. Conversely, by forming necks between the particles and strengthening the connections between them, raising the sintering temperature effectively improves the relative density. Additionally, it has been observed that higher sintering temperatures accelerate solid-state diffusion, which improves densification behavior^38,39^. For example, at a sintering temperature of 700 °C, the relative density of the samples SG0, SG1, SG2, SG4, and SG8 are 92.38, 91.81, 89.60, 87.77, and 84.89%, respectively, while the relative density of the same samples are 96.75, 96.54, 95.33, 94.06, and 92.41%, respectively. This trend can be more clarified using Eq. (11), in which it appears that the sintering temperature plays an essential role in the diffusion process^40^.

15 $${\text{D}} = {\text{D}}_{0} {\text{e}}^{{\frac{{ - {\text{Q}}}}{{{\text{RT}}}}}}$$

where *D*, *D*_0_, *Q*, *R*, and *T* represent for the diffusion coefficient, constant, activation energy, Boltzmann’s constant, and temperature, respectively.

### Morphology of the sintered nanocomposites

**Figure 7:**
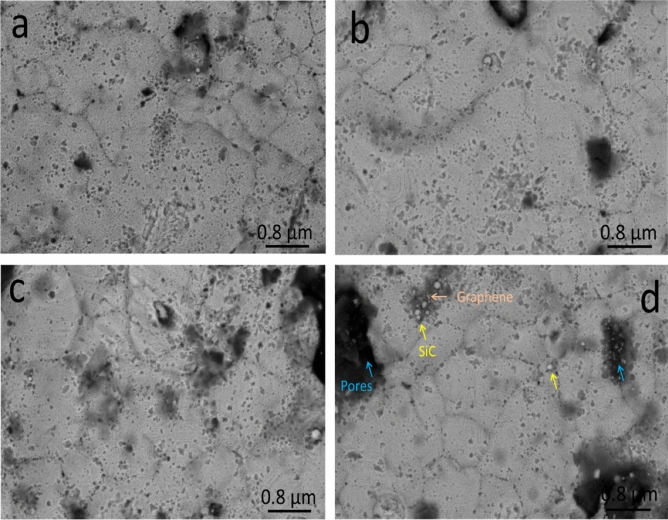
SEM images of (**a**) SG1, (**b**) SG2, (**c**) SG4, and (**d**) SG8 samples sintered at 850 °C.

**Figure 8:**
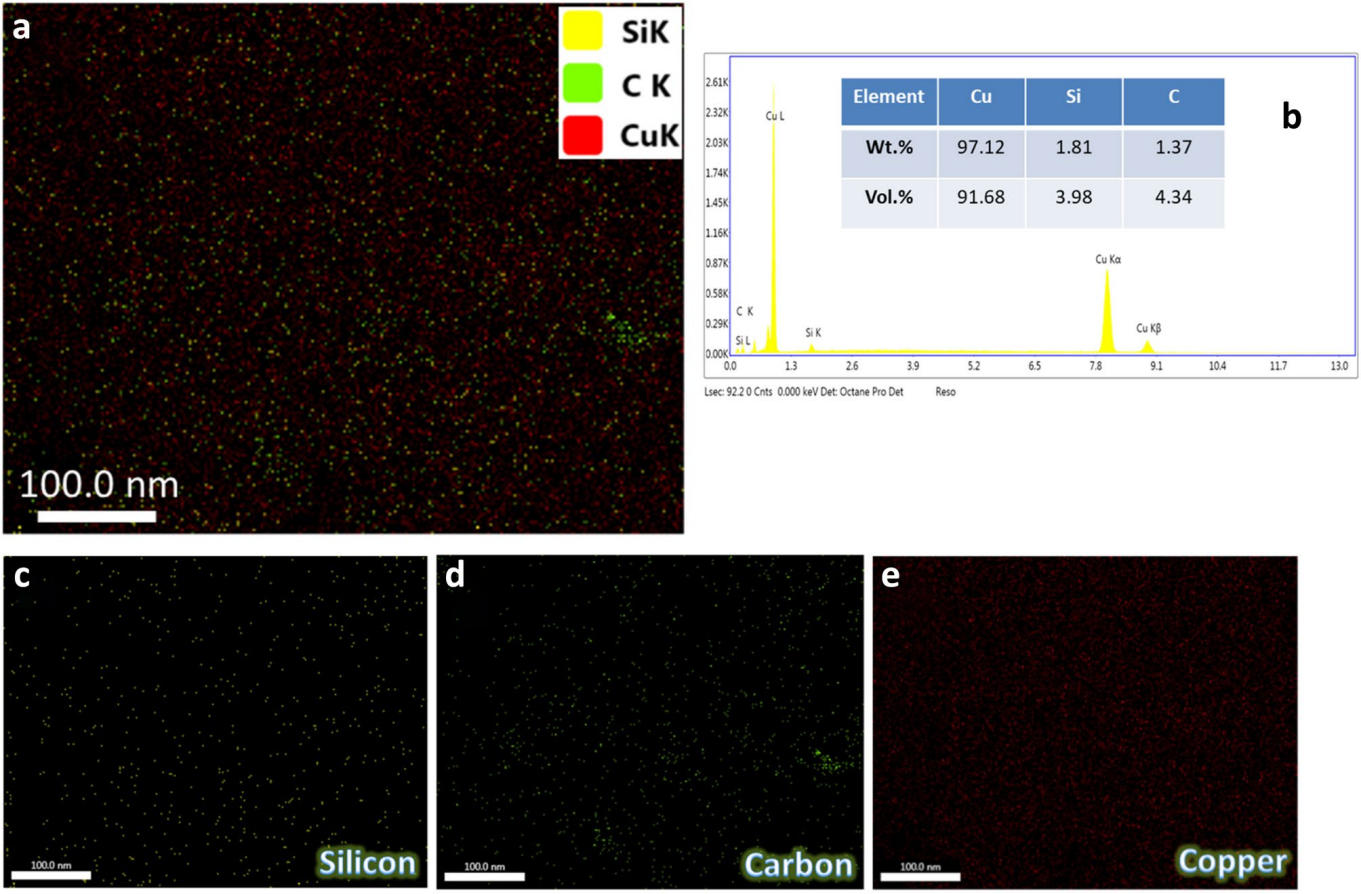
(**a**) EDX mapping of all constituents of GS8 sample.

Figure 7 illustrates the SEM images of SG1, SG2, SG4, and SG8 nanocomposites sintered at 850 °C for 1 h in an argon atmosphere. Generally, SiC nanoparticles and graphene nanosheets are found at the Cu matrix’s grain borders. As for samples SG1 and SG2, the amount of hybrid reinforcement is small, therefore not clearly visible, and more homogeneous. On the other hand, with an increase in the amount of hybrid reinforcements, as is evident in samples SG4 and SG8, it appears clearly with a decrease in homogeneity. Notably, it was also shown that in the specimens under study, the porosity rose as the number of reinforcing particles rose. Nevertheless, better densification behavior is produced by raising the sintering temperature to 850 °C, which promotes the diffusion process throughout the heating phase. Strong reinforcement matrix interfacial bonding was achieved during the sintering of the nanocomposites samples, as seen by the expanding contact border between the particles. Figure 8 shows the EDX spectrum and mapping of the distribution of each component in SC8 sample. Based on the data obtained, one can conclude that there are no other components, and thus the presence of contamination during milling or sintering processes can be ruled out. Another conclusion obtained from this figure is that: A well-homogeneous distribution of SiC nanoparticles and graphene nanosheets was obtained in Cu matrix.

### Thermal expansion

**Figure 9:**
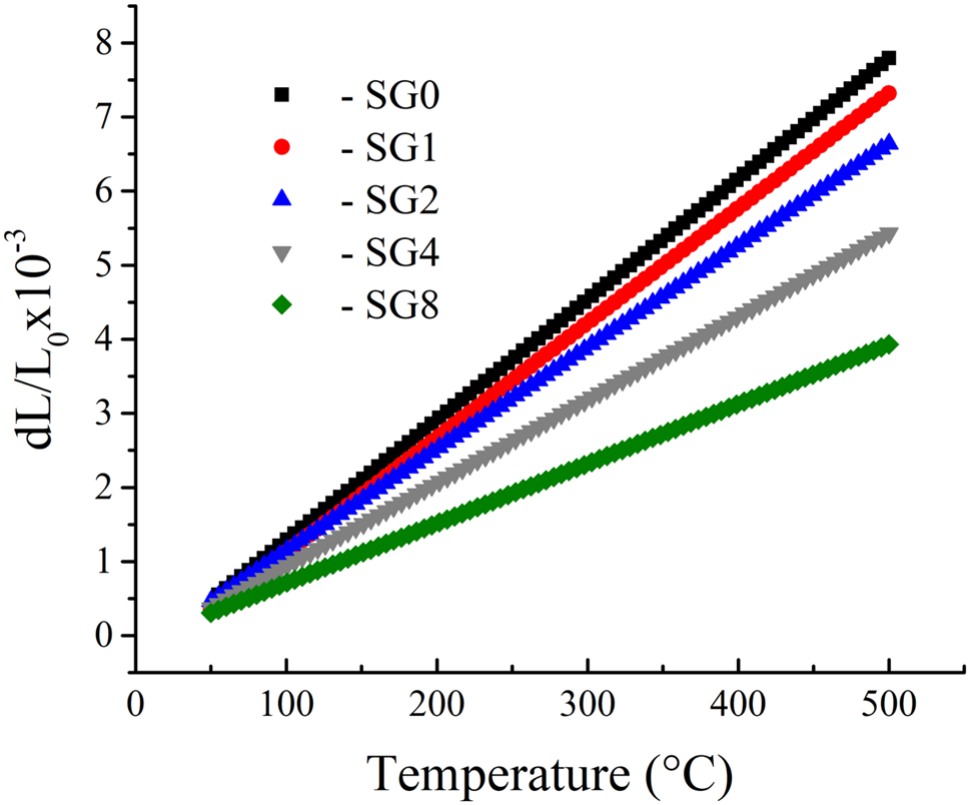
Relative thermal expansion of the sample sintered at 850 °C.

**Figure 10:**
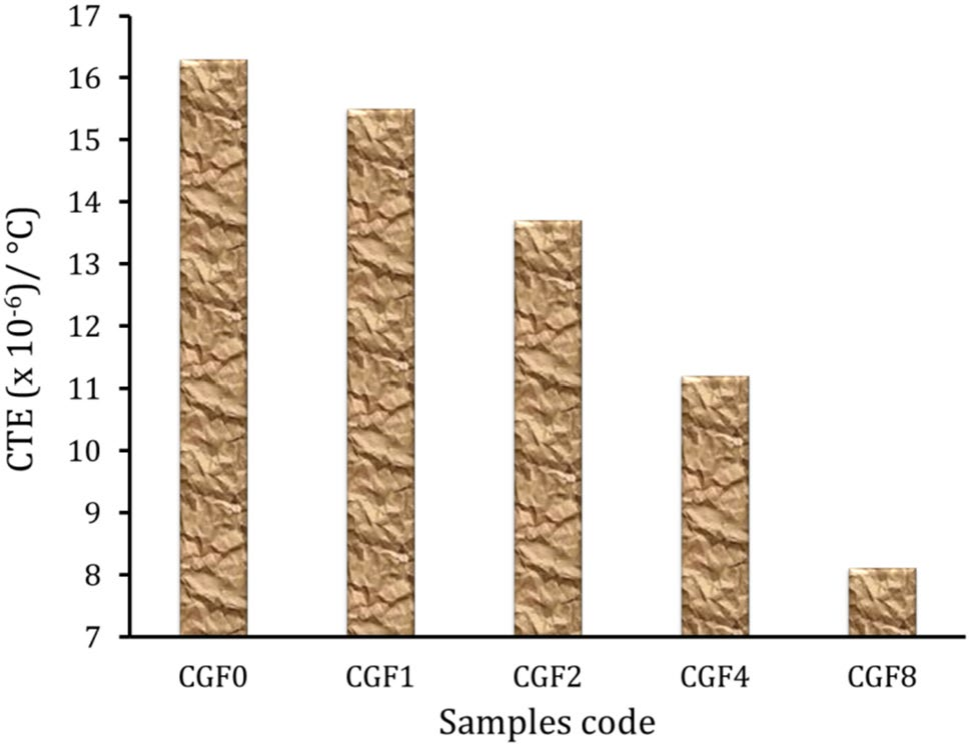
CTE value of the sample sintered at 850 °C.

The relative thermal expansion (ΔL/L) behavior of Cu and its nanocomposites was sintered at 850 °C at temperatures between 50 and 500 °C, as displayed in (Fig. 9). In general, as the temperature rose noticeably, the ΔL/L of the nanocomposites with different reinforcement concentrations increased. Additionally, it decreased as the amount of reinforcements increased. Figure 10 displays the CTE values of nanocomposites as calculated from the previous figure. The results showed that as the proportion of hybrid ceramics in the nanocomposites increased, the CTE value decreased. The CTE value for sample SG0 is 16.3 × 10^−6^/° C, which decreases to 15.9, 15, 13.6, and 11.4 × 10^−6^/° C for samples SG1, SG2, SG4, and SG8, respectively, which decreases by about 2.5, 8, 16.6, and 30.1% compared to the Cu matrix. The lower CTE of SiC and graphene (3.7 and 3.2 × 10^−6^/° C, respectively) than that of the Cu matrix (17 × 10^−6^/° C), which results from the bonding between the reinforcement and Cu matrix, is generally responsible for the lower CTE of the Cu matrix after the addition of hybrid reinforcements. Moreover, residual stresses arising from the thermal mismatch between the ceramics and the Cu matrix significantly influence the thermal expansion behavior of nanocomposite samples. The results agree with other literature^41–43^.

### Mechanical properties

**Figure 11:**
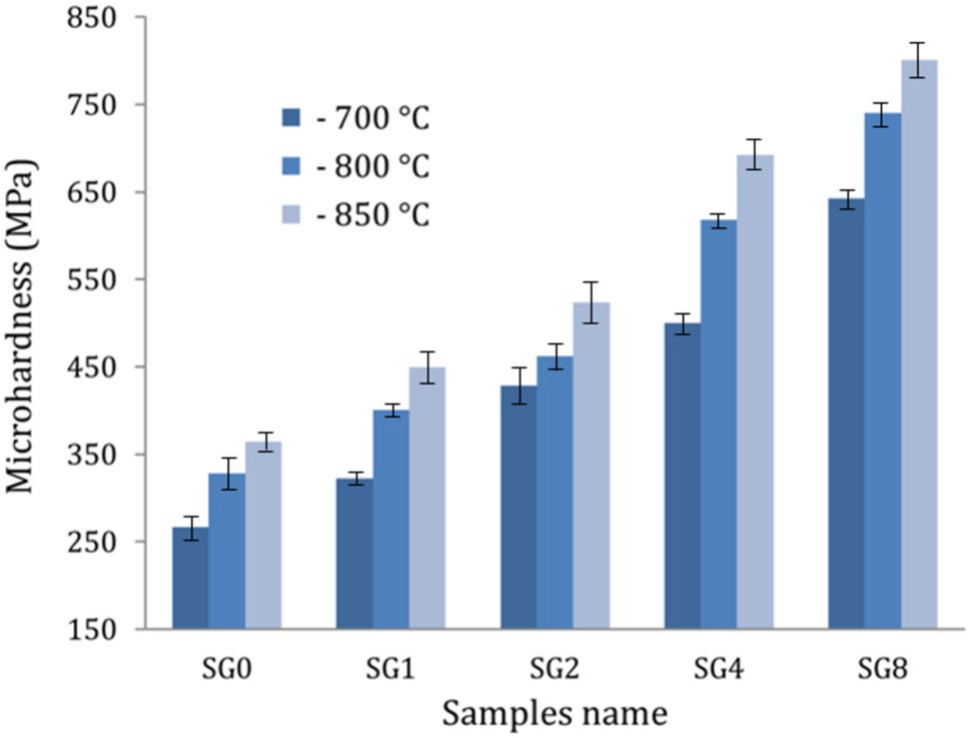
Microhardness of the samples at different sintering temperatures.

**Figure 12:**
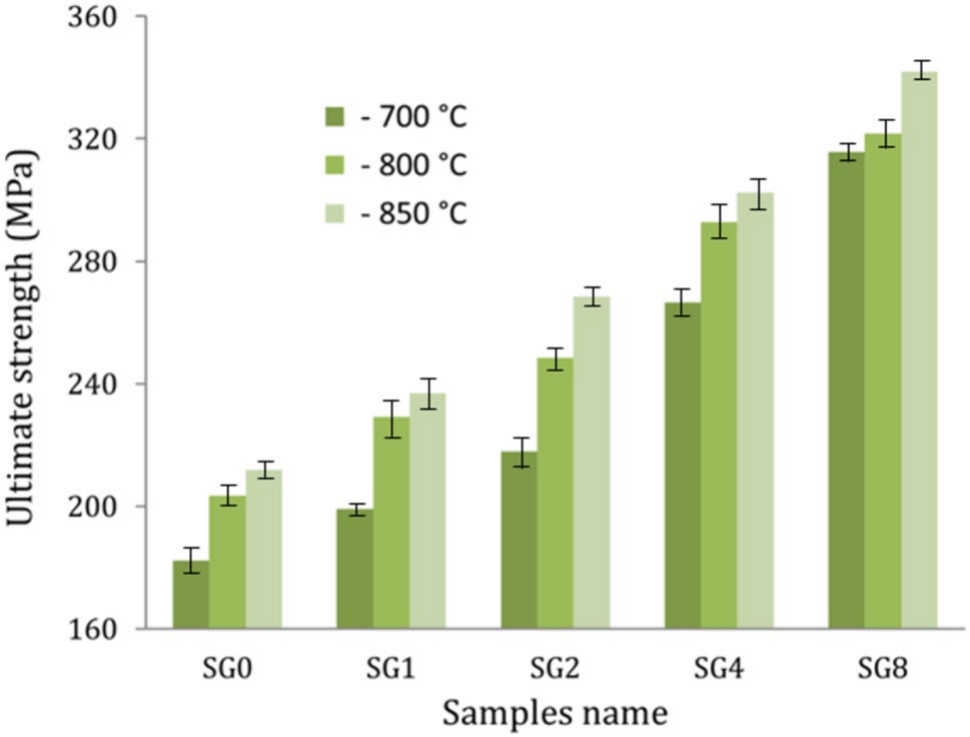
Ultimate strength of the samples at different sintering temperatures.

**Figure 13:**
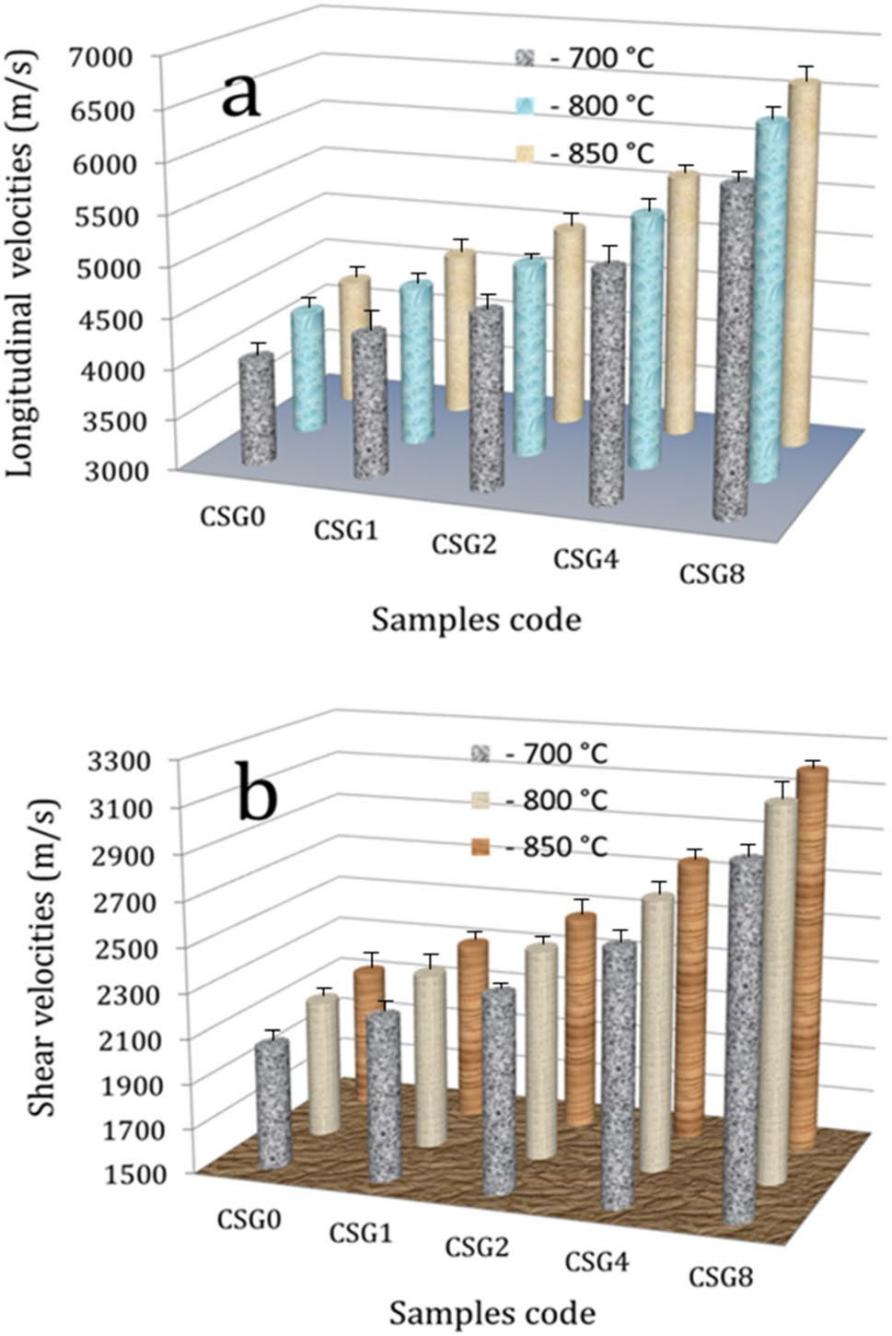
(**a**) Longitudinal and (**b**) shear ultrasonic velocities of all samples at different sintering temperatures.

Figures 11–13 show the effect of hybrid ceramics and sintering temperature on microhardness, ultimate strength, and ultrasonic velocities, while the values of elastic moduli are listed in Table 2 of the sintered samples. It is evident that there are significant improvements in microhardness, ultrasonic velocities, and elastic moduli after adding hybrid ceramics compared to Cu matrix. On the other hand, increasing the sintering temperature has a positive effect on improving the previous properties. The microhardness values of the SG0, SG1, SG2, SG4, and SG8 samples after sintering at 700 °C are 267.1, 317.8, 421.2, 496.6, and 639.7 MPa, respectively. At a sintering temperature of 850 °C, the microhardness values for the same samples increase to 364.1, 422.9, 498.7, 578.9, and 735.6 MPa, respectively. The longitudinal modulus values of the previous samples after sintering at 700 °C are 134.4, 157.5, 175.2, 207.5 and 262.6 GPa, respectively. At a sintering temperature of 850 °C, the values increase to 156.2, 183.1, 209.9, 256.5 and 336.6 GPa, respectively. The ultimate strength of the SG0, SG1, SG2, SG4, and SG8 samples after sintering at 700 °C is 182.2, 199.1, 217.9, 266.6, and 315.5 MPa, respectively. At a sintering temperature of 850 °C, the microhardness values for the same samples increase to 211.8, 236.9, 268.5, 302.4, and 341.8 MPa, respectively.

**Table 2:** The values of elastic moduli of nanocomposites sintered at 700, 800 and 850 °C.

Sample name	Temperature (°C)	Y (GPa)	L (GPa)	B (GPa)	S (GPa)	ν
SG0		91.68	134.42	99.88	34.54	0.3270
SG1		106.41	157.45	117.43	40.01	0.3296
SG2	700	117.38	175.21	131.15	44.06	0.3320
SG4		137.67	207.48	155.90	51.58	0.3346
SG8		170.91	262.47	198.67	63.80	0.3394
SG0		101.39	150.88	112.79	38.08	0.3312
SG1		116.91	175.75	131.93	43.84	0.3339
SG2	800	130.92	197.73	148.70	49.03	0.3352
SG4		154.78	237.19	179.39	57.80	0.3389
SG8		198.56	309.85	235.94	73.90	0.3434
SG0		104.11	156.22	117.18	39.04	0.3334
SG1		120.73	183.04	137.86	45.18	0.3361
SG2	850	137.08	209.87	158.67	51.20	0.3387
SG4		165.43	256.46	194.81	61.65	0.3418
SG8		212.08	336.56	257.86	78.70	0.3474

This noticeable improvement in the mechanical properties of the nanocomposites after adding hybrid ceramic is due to the good distribution of SiC nanoparticles and graphene nanosheets reinforcements in the Cu matrix, leading to the refinement of grain sizes and an increase in the number of grain boundaries, which act as barriers against dislocation movement^44,45^. This improvement can be better by observing this Eq. (14) Ref.^46^.

16 $${\text{H}}_{{\text{C}}} = {\text{H}}_{{{\text{Cu}}}} {\text{F}}_{{{\text{Cu}}}} + {\text{H}}_{{{\text{Graphene}}}} {\text{F}}_{{{\text{Graphene}}}} + {\text{H}}_{{{\text{SiC}}}} {\text{F}}_{{{\text{SiC}}}}$$

where, H_C_, H_Cu,_ H_C,_ H_Graphene and_ H_SiC_ represent the microhardness of the nanocomposite, Cu, graphene, and SiC, respectively, while F_Cu_, F_Graphene_ and F_SiC_ represent the volume fraction of Cu matrix, graphene nanosheets and SiC nanoparticles, respectively.

Moreover, a significant discrepancy in the CTE of the reinforcements (graphene nanosheet and SiC nanoparticle) and the Cu matrix contributes to the generation of thermally induced residual stresses. The thermal stresses produced in the Cu matrix, even at low temperatures, greatly increase the dislocation density close to the interface, strengthening the nanocomposite samples^47–49^. On the other hand, raising the sintering temperature causes an increase in atom diffusion, which expands the particle’s surface area and, as a result, raises the density and decreases the porosity of sintered nanocomposites.

### Wear behavior

**Figure 14:**
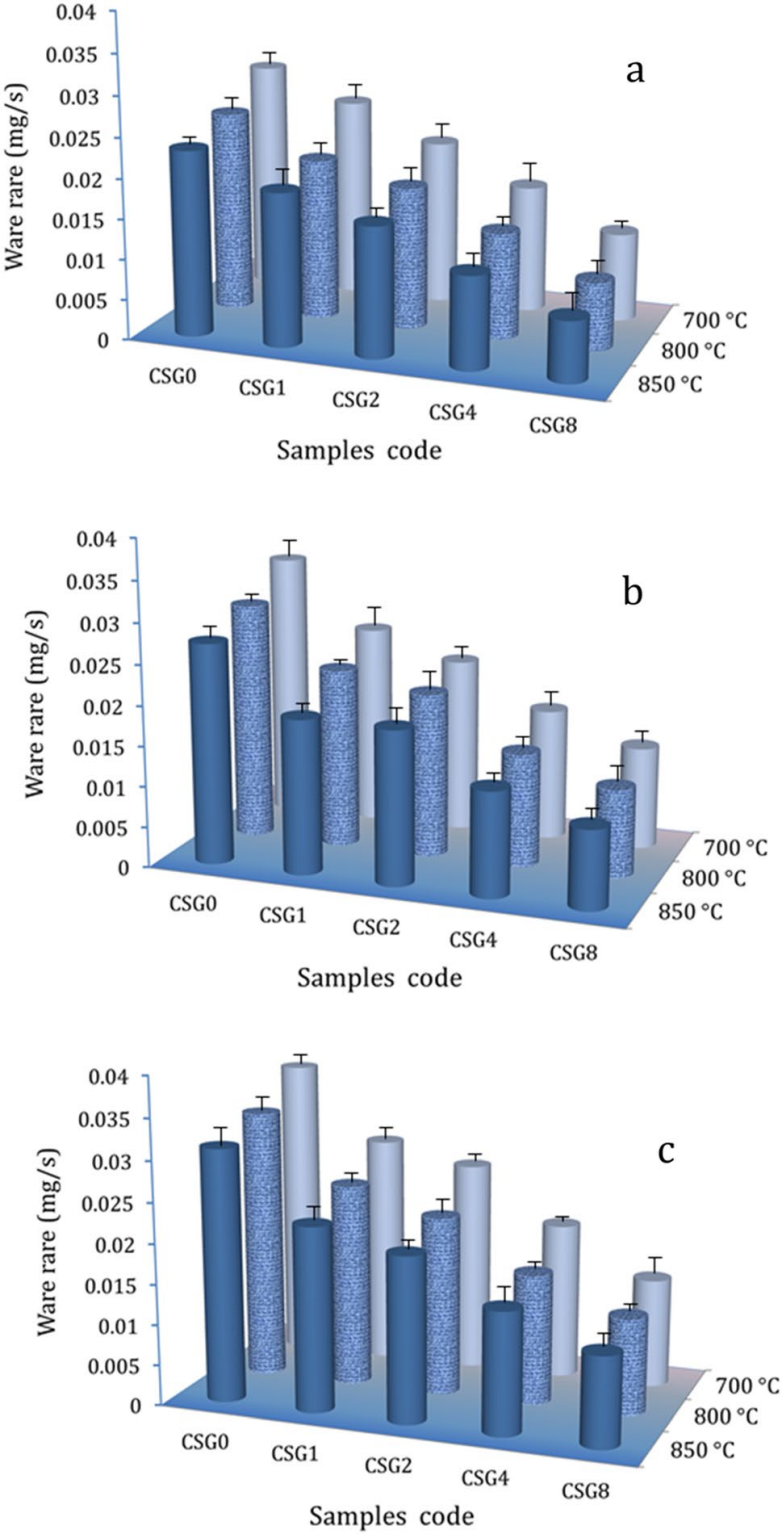
Wear rate of all samples sintered at 700, 800, and 850 °C under different applied: (**a**) 10 N, (**b**) 20 N, and (**c**) 40 N.

Table 3 illustrates how the applied load and hybrid reinforcement contents affect the weight loss of nanocomposite specimens sintered at 700, 800, and 850 °C for one hour. Interestingly, the table shows that significant increases in the weight loss of all samples under examination occur when the applied load is increased during sliding wear testing. On the other hand, raising the amount of hybrid ceramics and the sintering temperature decreases weight loss. Based on the finding that samples SG0, SG1, SG2, SG4, and SG8 sintered at 850 °C had determined weight loss values of 13.87, 8.98, 5.93, 4.22, and 2.76 mg, respectively, when the applied force equals 10 N. After increasing the applied load to 40 N, the weight loss for the same sample were 18.71, 13.69, 12.24, 9.01, and 6.65 mg, respectively. Figure 14 shows that the wear rate of nanocomposite samples was calculated using previous weight loss results. The results of the study led to the conclusion that adding hybrid reinforcements to nanocomposites and increasing the sintering temperature are more beneficial for wear resistance, while increasing the load is the opposite. The wear rate of SG1, SG2, SG4, and SG8 samples sintered at 700 °C was 0.0231, 0.0190, 0.0162, 0.0115, and 0.0075 mg/s, respectively, when the applied force equals 10 N which decreased by about 17.7, 30.1, 50.2, and 67.50%, respectively, compared to the SG0 sample (0.0231 mg/s). When the sintering temperature rises to 850 °C, The wear rate of previous samples was 0.0248, 0.0205, 0.0159, 0.0115, and 0.0110 mg/s, respectively, when the applied force equals 10 N which decreased by about 13, 28.20, 44.43, 61.48, and 6.65 mg, respectively, compared to the SG0 sample (0.0286 mg/s). It is essential to highlight that the addition of SiC nanoparticles and graphene nanosheets to the Cu matrix enhances the microhardness and strength of nanocomposites samples, as previously discussed, and as a result, the weight loss and wear rate decreases in accordance with Archad Eq. (16) Ref.^50^.

17 $${\text{W}} = \frac{{{\text{KP}}}}{{{\text{Hv}}}}$$

**Table 3:** The values of weight loss of nanocomposites sintered at different applied loads and sintering temperatures.

Sample name	Temperature (°C)	Weight loss (mg)		
		10 N	20 N	40 N
SG0		17.13	14.98	13.87
SG1		9.11	7.35	6.98
SG2	700	7.52	6.48	5.93
SG4		5.82	4.83	4.22
SG8		4.03	3.07	2.76
SG0		19.88	17.66	16.35
SG1		13.41	12.01	10.31
SG2	800	11.69	10.95	10.24
SG4		8.81	7.54	6.97
SG8		6.82	5.85	5.25
SG0		22.22	19.89	18.71
SG1		16.90	15.01	13.69
SG2	850	15.56	13.22	12.24
SG4		11.56	9.64	9.01
SG8		8.06	7.16	6.64

**Figure 15:**
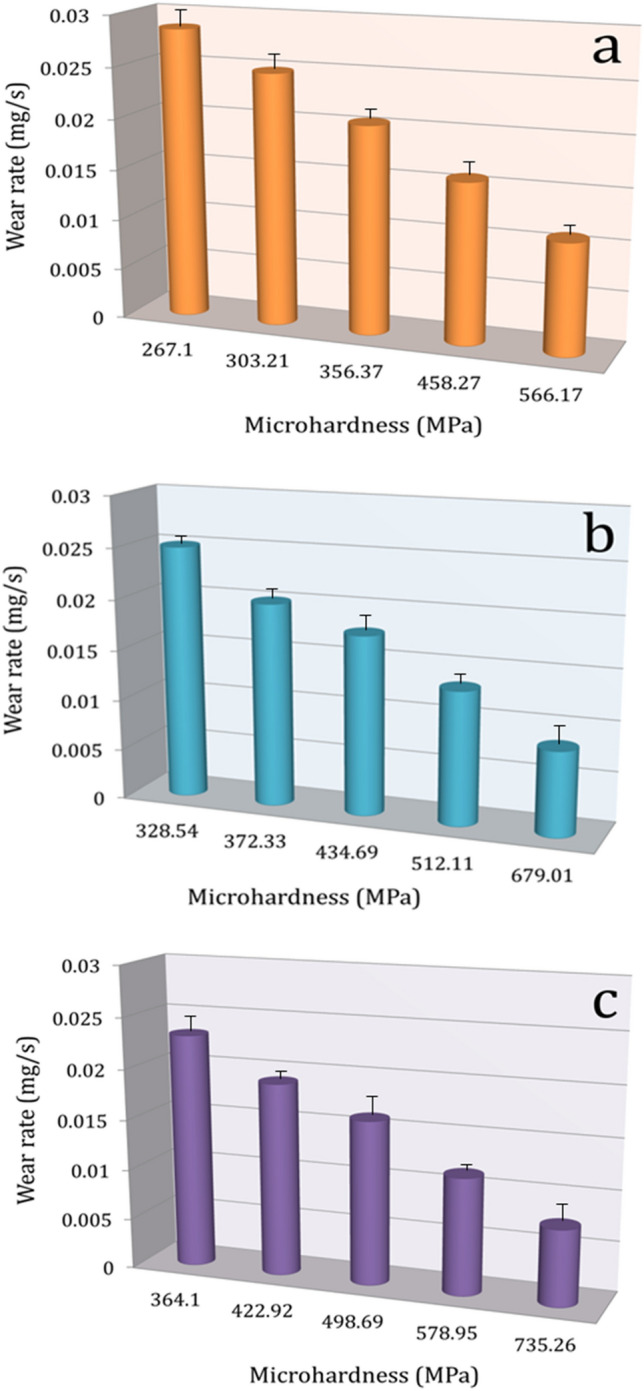
The effect of microhardness on the wear rate of the samples under load 10 N.

W is wear rate, K is a wear coefficient (constant value). This can be demonstrated by showing the effect of microhardness on the wear rate of the samples under load 10 N as shown in (Fig. 15). Moreover, a decrease in the actual area of contact is associated with an increase in microhardness. Reduced actual area of contact results in significant reductions in wear rate since it is widely acknowledged that real area of contact may be described as the ratio of the normal load to the hardness of the pin material^51,52^. On the other hand, the noticeable improvement in weight loss and wear rate with increasing sintering temperature is a result of the improvement in relative density, which is an important reason for the improvement in microhardness. Moreover, it is widely acknowledged that a rise in the load can result in a heating effect that causes convulsions and thermal softening. Moreover, it increases the surface area that slides when in contact, increasing the rate of wear^53^.

**Figure 16:**
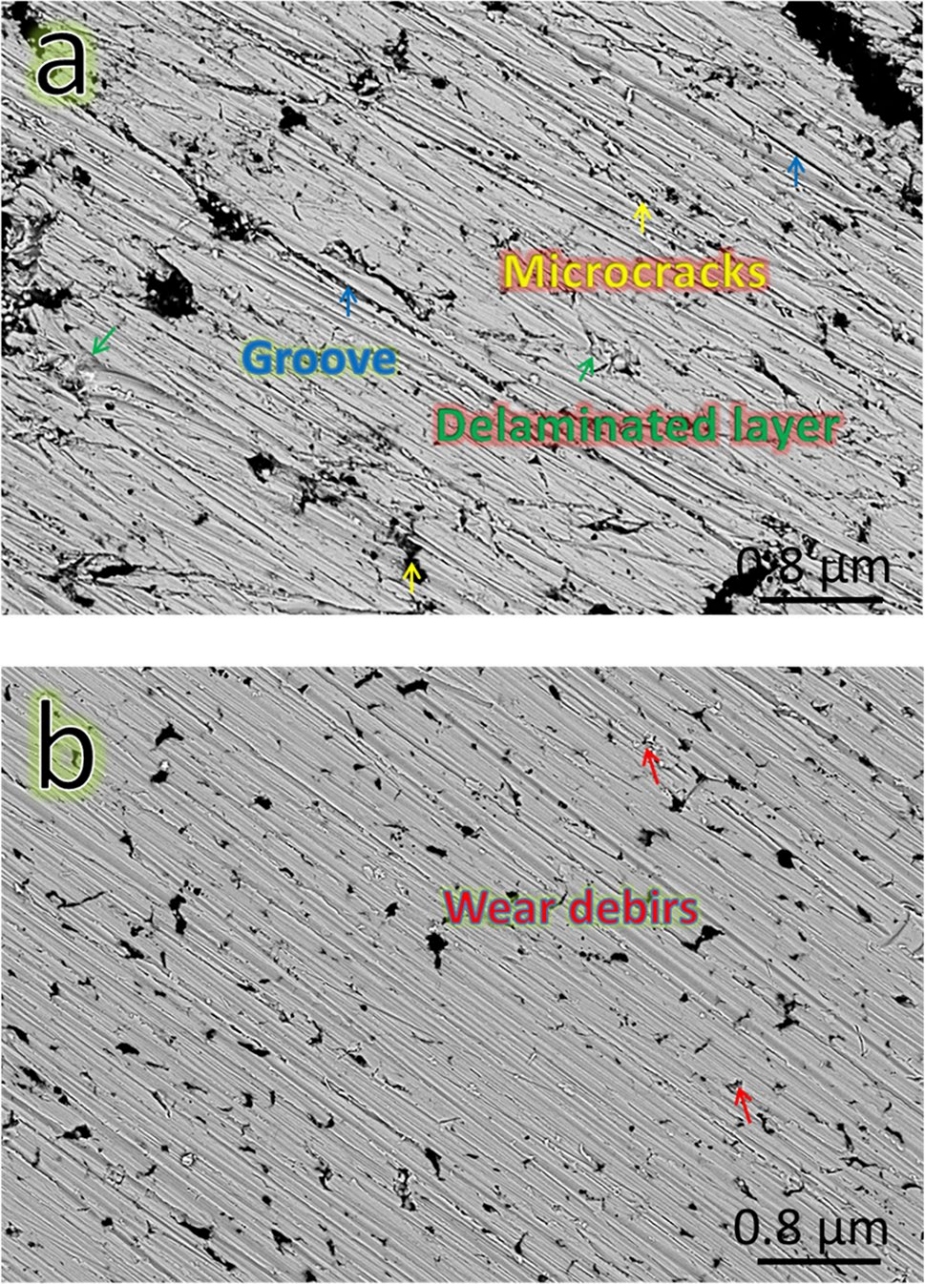
SEM images of wear tracks for (**a**) SG0 and (**b**) SG8 samples sintered at 850 and applied load 40 N.

Using FESEM measurements, the worn surfaces of the SG0 and SG8 samples were examined under an applied stress of 40 N to evaluate the wear processes of the Cu matrix and its nanocomposites, as shown in (Fig. 16a,b). For the Cu matrix, only loose layers and grooves appear on the wear track as shown in (Fig. 16a). Surface delamination reveals adhesive wear, which includes crack initiation and propagation as well as final fracture of the material in the vicinity of the surface. Figure 16b shows that the SG8 sample has a smoother surface than the SG0 sample, and there is only sporadic debris and slight grooves on the worn surface. Some Cu matrix debris has flattened in the wear process because of its low microhardness. Very low cracks appear in this wear track, thus, the dominant wear mechanism is abrasive wear.

### Electrical conductivity

**Figure 17:**
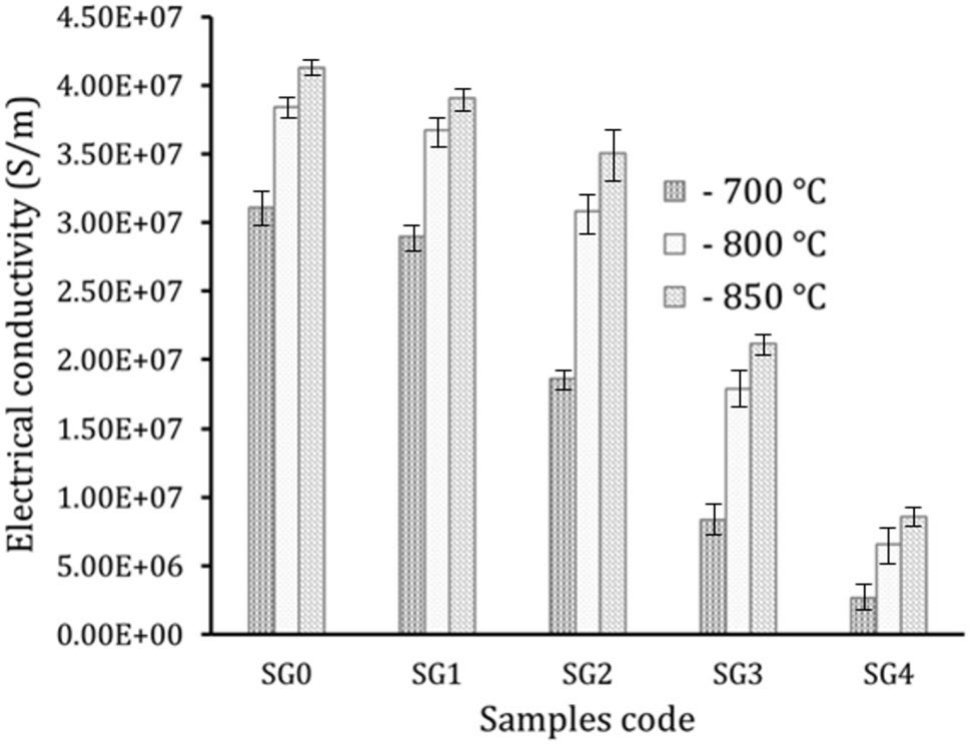
Electrical conductivity of the samples at different sintering temperatures.

The conductivity of each component, the amount, distribution, and size of reinforcement, the bulk density and Cu-reinforcements bonding, and the manufacturing process all unquestionably influence the electrical conductivity of nanocomposites. The electrical conductivity of sintered samples is shown in Fig. 17. Cu’s electrical conductivity somewhat reduces as the hybrid reinforcement’s volume percentage rises while increasing the sintering temperature has a positive effect on the electrical conductivity. Based on the finding that samples SG0, SG1, SG2, SG4 and SG8 samples sintered at 700 °C had determined electrical conductivity values of 3.11 × 10^7^, 2.98 × 10^7^, 1.86 × 10^7^, 8.38 × 10^6^ and 2.68 × 10^6^ S/m, respectively, while at sintering temperature 850 °C, the conductivity increases to 4.13 × 10^7^, 3.90 × 10^7^, 3.50 × 10^7^, 2.11 × 10^7^ and 8.59 × 10^6^ S/m, respectively. The apparent decrease in conductivity is due to the moving electrons into the structure is a major factor in Cu conductivity. Nevertheless, the addition of graphene nanosheets and SiC nanoparticles helped to distort this structure and impair the flow of electrons, which decreased the conductivity. Furthermore, refining the grains during the milling process increased the grain boundaries, hindering the movement of electrons^54^. In contrast, the noticeable increases in conductivity of samples with increasing sintering temperature are a result of the decrease the porosity.

## Conclusion

In this current study, the manufacturing of Cu-based hybrid nanocomposites with better mechanical properties, wear resistance, and CTE and using SiC nanoparticles and graphene nanosheets as reinforcements using the powder metallurgy (PM) technique was shown. After milling, the addition of hybrid reinforcement has an obvious effect on grain refinement, as the crystal and particle size decrease to 14.1 and 33.1 nm, respectively, for the sample containing high hybrid refinement contents (SG8). The finding showed that the addition of both hybrid reinforcements to the Cu base had a positive effect on improving mechanical properties, CTE value, and wear resistance and a slight negative effect on electrical conductivity. The apparent porosity of the nanocomposites increases with the addition of hybrid ceramics, while the relative density decreases.The maximum improved of microhardness, ultimate strength and bulk modulus about 122.1, 61.4, and 103.7%, respectably for the sample SG8 compared to Cu base (SG0). For the same sample, the maximum decrease in CTE value and wear rate about 50.2 and 80.13%, respectively Finally, increasing the sintering temperature has a wonderful effect on improving all properties, whether physical, mechanical, and electrical, wear resistance, and CTE value, as a result of improving the densification of the nanocomposites.

## Data Availability

The datasets generated and/or analyzed during the current study are not publicly available because all data are presented in the article and therefore, there is no need to include raw data but they are available from the corresponding author upon reasonable request.

## References

[1] Sulima, I. & Boczkal, G. Processing and properties of ZrB(2)-copper matrix composites produced by ball milling and spark plasma sintering. *Materials* **16**(23), 1–18 (2023). 10.3390/ma16237455 PMC10707533 38068198

[2] Akbarpour, M. R., Mirabad, H. M. & Gazani, F. An overview of friction stir processing of Cu–SiC composites: Microstructural, mechanical, tribological, and electrical properties. *Mater. Res. Technol.* **27**, 1317–1349 (2023). 10.1016/j.jmrt.2023.09.200

[3] Taha, M. A., El-zaidia, M. M., Zaki, M. Z. & Abomostafa, H. M. Influence of nano-hybrid reinforcements on the improvement strength, thermal expansion and wear properties of Cu–SiC–fly ash nanocomposites prepared by powder metallurgy. *ECS J. Solid State Sci. Technol.* **12**(3), 33011 (2023). 10.1149/2162-8777/acc5af

[4] Saber, D. *et al.* Characterization and performance evaluation of Cu-based/TiO(2) nano composites. *Sci. Rep. Apr.* **12**(1), 6669 (2023). 10.1038/s41598-022-10616-y PMC9035164 35461317

[5] Zawrah, M. F., Zayed, H. A., Essawy, R. A., Nassar, A. H. & Taha, M. A. Preparation by mechanical alloying, characterization and sintering of Cu–20 wt. % Al_2_O_3_ nanocomposites. *Mater. Des.* **46**, 485–490 (2013). 10.1016/j.matdes.2012.10.032

[6] Clinktan, R., Senthil, V., Ramkumar, K. R., Sivasankaran, S. & Al-Mufadi, F. A. Effect of boron carbide nano particles in CuSi4Zn14 silicone bronze nanocomposites on matrix powder surface morphology and structural evolution via mechanical alloying. *Ceram. Int.* **45**(3), 3492–3501 (2019). 10.1016/j.ceramint.2018.11.007

[7] Chen, F. *et al.* Effects of graphene content on the microstructure and properties of copper matrix composites. *Carbon* **96**, 836–842 (2016). 10.1016/j.carbon.2015.10.023

[8] Ahmed, M. A., Daoush, W. M. & El-Nikhaily, A. E. Fabrication and characterization of copper/silicon nitride composites. *Adv. mater. Res.* **5**(3), 131–140 (2016). 10.12989/amr.2016.5.3.131

[9] Akbarpoura, M. R., Mousa, M. H. & Alipour, S. Microstructural and mechanical characteristics of hybrid SiC/Cu composites with nano- and micro-sized SiC particles. *Ceram Int.* **45**, 3276–3283 (2019). 10.1016/j.ceramint.2018.10.235

[10] Somani, N. *et al.* Fabrication of Cu–SiC composites using powder metallurgy technique. *Mater. Today Proc.* **5**(14), 28136–28141 (2018). 10.1016/j.matpr.2018.10.055

[11] Priyaranjan, S., Harihar, T., Arabinda, M., Surekha, B. & Vundavilli, P. R. Effect of SiC and WC reinforcements on microstructural and mechanical characteristics of copper alloy-based metal matrix composites using sr casting, route. *Appl. Sci.* **13**, 1754 (2023). 10.3390/app13031754

[12] El-Zaidia, M. M., Zaki, M. Z., Abomostafa, H. M. & Taha, M. A. Comprehensive studies for evaluating promising properties of Cu/graphene/fly ash nanocomposites. *Sci. Rep.* **14**(1), 2235 (2024). 38278959 10.1038/s41598-024-52563-w PMC10817935

[13] Moustafa, E. B., Abdel Aziz, S. S., Taha, M. A. & Saber, A. H. Influence of graphene and silver addition on aluminum’s thermal conductivity and mechanical properties produced by the powder metallurgy technique. *Metals* **13**(5), 836 (2023). 10.3390/met13050836

[14] Fan, X. L., Ding, Q., Wang, H. & Hao, C. The microstructures and properties of in-situ ZrB_2_ reinforced Cu matrix composites. *Res. Phys.* **14**, 102494 (2019).

[15] Guo, S. *et al.* In situ synthesis of high content graphene nanoplatelets reinforced Cu matrix composites with enhanced thermal conductivity and tensile strength. *Powder. Technol.* **362**, 126–134 (2020). 10.1016/j.powtec.2019.11.121

[16] Singh, M. K. & Gautam, R. K. Structural, mechanical, and electrical behavior of ceramic-reinforced copper metal matrix hybrid composites. *J. Mater. Eng. Perform.* **28**, 886–899 (2019). 10.1007/s11665-019-3860-x

[17] Akbarpoura, M. R., MousaMirabada, H. & Alipour, S. Microstructural and mechanical characteristics of hybrid SiC/Cu composites with nano- and micro-sized SiC particles. *Ceram. Int.* **45**, 3276–3283 (2019). 10.1016/j.ceramint.2018.10.235

[18] Yadav, P., Dwivedi, S. P., Shahnawaz, M., Singh, A. & Yadav, J. Development of copper based composite by stir casting technique. *Mater. Today Proc.* **25**, 649–653 (2020). 10.1016/j.matpr.2019.07.669

[19] Chebolu, R., Nallu, R., Chanamala, R., Sharma, S. K. & Rudrapati, R. Influence of SiC/TiB particles addition on corrosion behavior of as-cast zn-al-cu alloy hybrid composites. *J. Eng.* **22**, 3669584 (2022).

[20] Khoshaim, A. B., Moustafa, E. B., Alazwari, M. A. & Taha, M. A. An investigation of the mechanical, thermal and electrical properties of an AA7075 aloy reinforced with hybrid ceramic nanoparticles using friction stir processing. *Metals* **13**(1), 124 (2023). 10.3390/met13010124

[21] Ramkumara, K. R., Radhika, N., Sivasankaranc, S. & Kim, H. S. Evolution of microstructure and mechanical properties of [Cu–10Ni]–Si_3_N_4_ nanocomposites developed using mechanical alloying and spark plasma sintering. *Alloy. Compd.* **899**, 163319 (2022). 10.1016/j.jallcom.2021.163319

[22] Yan, Y. F. *et al.* Ceramic particles reinforced copper matrix composites manufactured by advanced powder metallurgy: Preparation, performance, and mechanisms. *Int. J. Extr. Manufact.* **5**(3), 2241 (2023).

[23] Moustafa, E. B. *et al.* Fabrication and characterization of functionally graded nanocomposites: Impact of graphene and vanadium carbide on aluminum matrix. *ECS J. Solid State Sci. Technol.* **13**, 053012 (2024). 10.1149/2162-8777/ad4c96

[24] Youness, R. A., Zawrah, M. F. & Taha, M. A. Fabrication of akermanite scaffolds with high bioactivity and mechanical properties suitable for bone tissue engineering application. *Ceram. Int.*10.1016/j.ceramint.2024.06.033 (2024). 10.1016/j.ceramint.2024.06.033

[25] Youness, R. A., Saleh, H. A. & Taha, M. A. Microstructure and elastic properties of hydroxyapatite/alumina nanocomposites prepared by mechanical alloying technique for biomedical applications. *Biointerface. Res. Appl. Chem.* **13**(4), 395 (2023).

[26] Abushanab, W. *et al.* Influence of vanadium and niobium carbide particles on the mechanical, microstructural, and physical properties of AA6061 aluminum-based mono- and hybrid composite using FSP. *Coatings* **13**(1), 142 (2023). 10.3390/coatings13010142

[27] Sadek, H. E. H. *et al.* Effect of ZnO, TiO_2_ and Fe_2_O_3_ on inhibition of spinel formation during cordierite fabrication: Sinterability, physico-mechanical and electrical properties. *J. Inorg. Organomet. Polym. Mater.* **34**(3), 1068–1080 (2023). 10.1007/s10904-023-02750-5

[28] Elwan, R. L., El-Kheshen, A. A., Youness, R. A. & Taha, M. A. Exploitation of ladle furnace iron slag for semiconductor borosilicate glass production. *Ceram. Int.* **49**(23), 37680–37690 (2023). 10.1016/j.ceramint.2023.09.094

[29] Fathi, A. M., Fayad, A. M., El-Beih, A. A., Taha, M. A. & Abdel-Hameed, S. A. M. Effect of ZnO addition on structure, bioactivity, and corrosion protection of mica-fluorapatite glass/glass-ceramic. *J. Aust. Ceram. Soc.* **57**, 1241–1253 (2021). 10.1007/s41779-021-00618-w

[30] Youness, R. A. & Taha, M. A. Tuning biodegradability, bone-bonding capacity, and wear resistance of zinc-30% magnesium intermetallic alloy for use in load-bearing bone applications. *Sci. Rep.* **14**(1), 2425 (2024). 38287092 10.1038/s41598-024-52648-6 PMC10825179

[31] Alturki, A. M. *et al.* Magnetic and dielectric properties of hybrid nanocomposites of biologically extracted hydroxyapatite/hematite/silicon dioxide for potential use in bone replacement applications. *ECS J. Solid State Sci. Technol.* **12**(8), 083001 (2023). 10.1149/2162-8777/ace994

[32] Abulyazied, D. E. *et al.* Production of polyvinyl alcohol/natural hydroxyapatite/magnesia/silicon carbide hybrid composites for use in orthopedic applications: Optical, electrical, and mechanical properties. *Egypt. J. Chem.* **68**(11), 411–422 (2024).

[33] Youness, R. A., Al-Ashkar, E. & Taha, M. A. Role of porosity in the strength, dielectric properties, and bioactivity of hardystonite ceramic material for use in bone tissue engineering applications. *Ceram. Int.* **49**(24), 40520–40531 (2023). 10.1016/j.ceramint.2023.10.029

[34] Abushanab, W. S., Moustafa, E. B. & Youness, R. A. Mechanical behavior and tribological properties of hydroxyapatite/hardystonite/zirconia hybrid nanocomposites for orthopedic applications. *Appl. Phys. A* **129**(6), 394 (2023). 10.1007/s00339-023-06675-1

[35] Abushanab, W. S. *et al.* Impact of hard and soft reinforcements on the microstructure, mechanical, and physical properties of the surface composite matrix manufactured by friction stir processing. *Coatings* **13**(2), 284 (2023). 10.3390/coatings13020284

[36] Chu, K. & Jia, C. Enhanced strength in bulk graphene–copper composites. *Phys. stat. solid* **211**(1), 184–190 (2013).

[37] Du, X., Zheng, K. & Liu, F. Microstructure and mechanical properties of graphene-reinforced aluminum-matrix composites. *Mater. Technol.* **52**(6), 763–768 (2018).

[38] Ağaoğullari, D. Effects of ZrC content and mechanical alloying on the microstructural and mechanical properties of hypoeutectic Al-7 wt. % Si composites prepared by spark plasma sintering. *Ceram. Int.* **45**(10), 13257–13268 (2019). 10.1016/j.ceramint.2019.04.013

[39] Wahi, A., Muhamad, N., Sulon, A. B. & Ahmad, R. N. Effect of sintering temperature on density, hardness and strength of MIM Co30Cr6Mo biomedical alloy. *J. Jpn. Soc. Powder. Metall.* **63**, 434–437 (2016). 10.2497/jjspm.63.434

[40] Moustafa, E. B. & Taha, M. A. Evaluation of the microstructure, thermal and mechanical properties of Cu/SiC nanocomposites fabricated by mechanical alloying. *Int. J. Miner. Metall. Mater.* **28**(3), 475–486 (2021). 10.1007/s12613-020-2176-z

[41] Lei, Z., Zhao, K., Wang, Y. & An, L. Thermal expansion of Al matrix composites reinforced with hybrid micro-/nano-sized Al_2_O_3_ particles. *Mater. Sci. Technol.* **30**(1), 61–64 (2014). 10.1016/j.jmst.2013.04.022

[42] Kamardin, A., Derman, M. N. & Bakri, A. M. M. A. The themal expansion behavior of Cu–SiCp composites. *Adv. Mater. Res.* **795**, 237–240 (2013). 10.4028/www.scientific.net/AMR.795.237

[43] Moustafa, E. B., AbuShanab, W. S., Youness, R. A. & Taha, M. A. Improved mechanical properties of Cu8Ni4Sn alloy as functionally graded composites with preserving its thermal and electrical properties. *Mater. Chem. Phys.* **292**, 126778 (2022). 10.1016/j.matchemphys.2022.126778

[44] Moustafa, E. B. *et al.* A comprehensive study of Al–Cu–Mg system reinforced with nano-ZrO_2_ particles synthesized by powder metallurgy technique. *Sci. Rep.* **14**(1), 2862 (2024). 38311645 10.1038/s41598-024-53061-9 PMC10838939

[45] Balasundar, P., Senthil, S., Narayanasamy, P. & Ramkumar, T. Microstructure and tribological properties of microwave-sintered Ti0.8Ni–0.3Mo/TiB composites. *Ceram. Int.* **49**(4), 6055–6062 (2023). 10.1016/j.ceramint.2022.11.085

[46] Samal, P., Tarai, H., Meher, A., Surekha, B. & Vundavilli, P. R. Effect of SiC and WC reinforcements on microstructural and mechanical characteristics of copper alloy-based metal matrix composites using stir casting route. *Appl. Sci.* **13**(3), 1754 (2023). 10.3390/app13031754

[47] Balasundar, P., Senthil, S., Narayanasamy, P. & Ramkumar, T. Mechanical, thermal, electrical, and corrosion properties of microwave-sintered Ti-0.8Ni-0.3Mo/TiB composites. *Phys. Scr* **98**(6), 065954 (2023). 10.1088/1402-4896/acd6c5

[48] Moustafa, E. B., Abushanab, W. S., Ghandourah, E. I., Taha, M. A. & Mosleh, A. O. Advancements in surface reinforcement of AA2024 alloy using hybridized niobium carbide and ceramics particles via FSP technique. *Met. Mater. Int.* **30**, 800–813 (2023). 10.1007/s12540-023-01541-4

[49] Lu, T. *et al.* W and TiO_2_ particles synergistic strengthened Cu matrix nanocomposites by mechano-chemical method. *Powder. Metall.* **5**, 381–391 (2020). 10.1080/00325899.2020.1833139

[50] AbuShanab, W. S., Moustafa, E. B., Ghandourah, E. & Taha, M. A. The effect of different fly ash and vanadium carbide contents on the various properties of hypereutectic Al–Si alloys-based hybrid nanocomposites. *Silicon* **14**(10), 5367–5377 (2022). 10.1007/s12633-021-01284-0

[51] Tyagi, H. Effect of TiC content on friction and wear behavior of Al-tic composites. *Wor. Tribol. Cong.* **1**, 3–4 (2005).

[52] Idusuyi, N. & Olayinka, J. I. Dry sliding wear characteristics of aluminium metal matrix composites: A brief overview. *J. Mater. Res. Technol.* **8**(3), 3338–3346 (2019). 10.1016/j.jmrt.2019.04.017

[53] Moustafa, E. B. & Taha, M. A. The effect of mono and hybrid additives of ceramic nanoparticles on the tribological behavior and mechanical characteristics of an Al-based composite matrix produced by friction stir processing. *Nanomaterials* **13**(14), 2148 (2023). 37513159 10.3390/nano13142148 PMC10385158

[54] Moustafa, E. B., Abushanab, W. S., Ghandourah, E. I., Taha, M. A. & Mosleh, A. O. Advancements in surface reinforcement of AA2024 alloy using hybridized niobium carbide and ceramics particles via FSP technique. *Met. Mater. Int.* **30**(3), 800–813 (2023). 10.1007/s12540-023-01541-4

